# Inferring Fitness Effects from Time-Resolved Sequence Data with a Delay-Deterministic Model

**DOI:** 10.1534/genetics.118.300790

**Published:** 2018-03-02

**Authors:** Nuno R. Nené, Alistair S. Dunham, Christopher J. R. Illingworth

**Affiliations:** * Department of Genetics, University of Cambridge, CB2 3EH, UK; † Department for Applied Mathematics and Theoretical Physics, Centre for Mathematical Studies, University of Cambridge, Cb3 OWA, UK

**Keywords:** inference of fitness landscapes, time-resolved sequence data, delay-deterministic model, viral adaptation

## Abstract

A common challenge arising from the observation of an evolutionary system over time is to infer the magnitude of selection acting upon a specific genetic variant, or variants, within the population. The inference of selection may be confounded by the effects of genetic drift in a system, leading to the development of inference procedures to account for these effects. However, recent work has suggested that deterministic models of evolution may be effective in capturing the effects of selection even under complex models of demography, suggesting the more general application of deterministic approaches to inference. Responding to this literature, we here note a case in which a deterministic model of evolution may give highly misleading inferences, resulting from the nondeterministic properties of mutation in a finite population. We propose an alternative approach that acts to correct for this error, and which we denote the delay-deterministic model. Applying our model to a simple evolutionary system, we demonstrate its performance in quantifying the extent of selection acting within that system. We further consider the application of our model to sequence data from an evolutionary experiment. We outline scenarios in which our model may produce improved results for the inference of selection, noting that such situations can be easily identified via the use of a regular deterministic model.

## Abstract (precis)

A broad range of approaches have considered the challenge of inferring selection from time-resolved genome sequence data. Models describing deterministic changes in allele or haplotype frequency have been highlighted as providing accurate and computationally...

FITNESS landscapes describe the relationship between the genome of an organism and its evolutionary fitness (de Visser and Krug 2014). Evolutionary fitness encompasses a broad range of important phenotypes of an organism, making the inference of details of fitness landscapes a topic of broad biological interest. In some important biological systems, adaptation occurs as a rapid and ongoing process (Buonagurio *et al.* 1986; Bergland *et al.* 2014). Where multiple beneficial mutations arise in a population simultaneously, linkage between mutations has a substantial impact upon evolutionary processes; a considerable body of literature has characterized the implications of such effects for adaptation (Barton 1995; Gerrish and Lenski 1998; Gillespie 2001; Rouzine *et al.* 2003; Desai and Fisher 2007; Schiffels *et al.* 2011; Good *et al.* 2012; Rouzine and Weinberger 2013).

Where adaptation is sufficiently rapid to be observed, time-resolved sequence data may be of assistance in measuring the extent to which a variant is under selection. Under the assumption of a large population size, the evolution of a single beneficial allele over time can be described by deterministic differential equations (Hartl and Clark 2007). Given sufficient observations of a population under study, the simplicity of this deterministic framework allows it to be extended to infer selection in far more complicated evolutionary scenarios (Illingworth and Mustonen 2011, 2013); fitting a deterministic model to data provides an estimate of the magnitude of selection acting upon one or very many alleles. In other situations, genetic drift is an important factor to account for; in a small population, changes in allele frequency occurring via drift may outweigh those caused by selection (Rouzine *et al.* 2001). In this situation, a variety of methods have therefore been developed to consider the evolution of a single-locus, two-allele system, estimating in a joint calculation the effective size of a population, and the magnitude of selection acting upon a variant allele (O’Hara 2005; Bollback *et al.* 2008; Malaspinas *et al.* 2012; Mathieson and McVean 2013; Foll *et al.* 2014; Lacerda and Seoighe 2014; Ferrer-Admetlla *et al.* 2016; Schraiber *et al.* 2016). In a similar calculation, one may estimate whether or not a change in the frequency of an allele has arisen through selection or genetic drift. Genetic drift induces an uncertainty in the future frequency of an allele (Kimura 1955); accounting for this, alleles that have changed by more than a given threshold may be identified, enabling the attribution of selection to genetic variants (Feder *et al.* 2014; Terhorst *et al.* 2015; Topa *et al.* 2015). A similar approach has been applied to the case where a population is large, but measurements of allele frequency are of limited quality; model selection procedures discriminate “neutral” from “selected” behavior in an allele frequency trajectory (Illingworth *et al.* 2014).

Where genetic drift is incorporated into a model, a variety of approaches to modeling Wright–Fisher propagation have been adopted (Tataru *et al.* 2016). Numerical solution of the stochastic dynamics of the population may be computationally intensive, inspiring the development of more rapid propagation methods and the consideration of potential alternative solutions (Khatri 2016; Krukov *et al.* 2017; Nené *et al.* 2018). In a recent work, considering a range of potential models for the demographic history of a population, it was concluded that deterministic approximations to evolution under drift can produce accurate estimates of the magnitude of selection (Jewett *et al.* 2016). Such models of selection, mutation, and recombination have been used to generate insights into viral adaptation (Ganusov *et al.* 2011; Illingworth 2015; Sobel Leonard *et al.* 2017). Time-resolved sequence data describing pathogenic populations is becoming increasingly available (Shankarappa *et al.* 1999; Zanini *et al.* 2015; Houldcroft *et al.* 2017; Xue *et al.* 2017); in so far as demographic effects can be ignored in such systems, evolutionary inference becomes possible at far-reduced computational cost, making this an important area for methodological development and application.

While acknowledging the potential for deterministic models to generate biological insight, we here present an important case in which a deterministic inference of selection from population sequence data produces a severely deficient result. In this case, a stochastic approach to inference produces a correct result, albeit with additional prior knowledge of the system and at the cost of a substantial amount of computational time. However, the use of what we term a delay-deterministic model, including a single extra model parameter, goes a substantial way to correcting the error in the deterministic calculation. We propose that under a range of evolutionary circumstances, the delay-deterministic model provides a useful framework for inference, combining the speed of a deterministic modeling framework with the accuracy achievable by more computationally intensive models.

## Materials and Methods

### Simulated trajectories under a Wright–Fisher propagation model

Simulated data from a population was generated according to a model of sequential mutation and selection steps. In this study, we wish to consider effects that arise when a population evolves into new haplotypes via mutation and positive selection; mutation creating individuals with new and fitter genotypes that then grow as a fraction of the population under the influence of positive selection. Such patterns of evolution have been observed in the experimental adaptation of an influenza virus to a novel mammalian host (Imai *et al.* 2012; Wilker *et al.* 2013). This imposes strong selection upon the virus; given the large size of a within-host influenza population and the high mutation rate of the virus, successive beneficial mutations may be gained relatively quickly.

**Figure 1:**
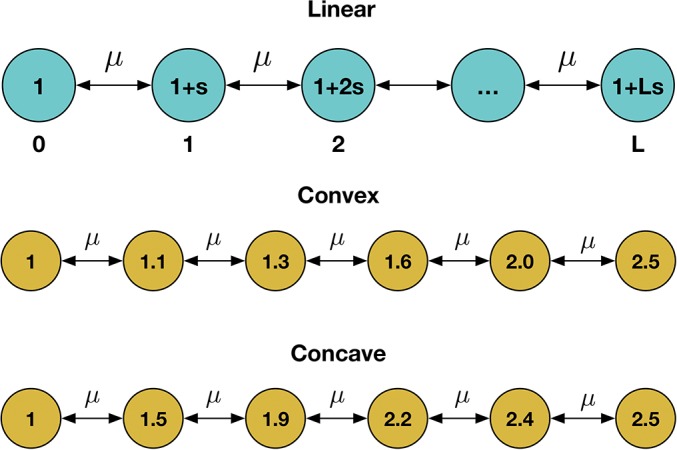
Linear haplotype models used for simulation. In the linear model, the $i^{th}$ haplotype has fitness $w_{i}=1+is.$ Convex and concave models (in which the fitness differences between haplotypes either increase or decrease with distance from the original haplotype) were also tested. In all models, mutation occurs between adjacent haplotypes with constant rate *μ*.

In this study, we consider a simplified version of this model, comprising a population of *N* individuals occupying a linear network of L+1 distinct haplotypes, each haplotype being separated from the previous one by a single mutation. We model the fitness of each haplotype as continually increasing with the gain of each successive mutation, such that the fitness of haplotype *i* is given by $w_{i}=1+\sum_{j=1}^{i}s_{j}$ for some arbitrary set of parameters $s_{j}>0.$ In this study, we consider populations with a linear gain in fitness (*i.e.*, with the restriction $s_{j}=s$ for all *j*), and examples of convex and concave fitness landscapes, for which this restriction does not apply, the gain of fitness with each additional haplotype either increasing or decreasing with the gain of each successive mutation (Figure 1). For reasons of computational efficiency, we restrict our model to systems with up to six distinct haplotypes.

Within a simulated population, we denote the number of individuals of haplotype *i* in the population after *t* generations by $n_{i}\left(t\right).$ Each generation, propagation of the system was conducted using a simple model of mutation and selection. Mutation was modeled as occurring between adjacent haplotypes; for a given mutation rate *μ*, the number of mutants $m_{ij}$ from haplotype *i* into an adjacent haplotype *j* was described by a Poisson distribution

$$P\left(m_{ij}=k\right)=\frac{{\left(\mu n_{i}\left(t\right)\right)}^{k}}{k!}e^{-\mu n_{i}\left(t\right)}$$ (1)

Subsequently, the next generation was drawn via a multinomial process

$$P\left(n\left(t+1\right)\right)=\frac{N!}{\prod_{i}n_{i}\left(t+1\right)!}\prod_{i=1}^{L}{\left(\frac{{\tilde{n}}_{i}\left(t\right)w_{i}}{N\bar{w}}\right)}^{n_{i}\left(t+1\right)}$$ (2)

where $w_{i}$ is the fitness of the haplotype *i*, ${\tilde{n}}_{i}\left(t\right)$ is the number of individuals of haplotype *i* at time *t* after the effect of mutation has been accounted for, and $\bar{w}$ is the mean fitness of the population. Sequencing of the population was simulated via a multinomial emission model with sequencing read depth $N_{d}.$ So as to understand the performance of our inference model, we considered versions of the linear system with a variety of parameters. Simulations were conducted with $\mu =10^{-5},$ population size $N\in \left\{10^{5},10^{9}\right\},$ and s∈{0.1,0.2,…,0.9}. Samples from the population were collected via a multinomial process at the times $\left\{t_{k}\right\}$ for k=0,…,K, with sampling ceasing as soon as 99% of the population occupied the last, and fittest, haplotype in the system. By default, sampling was conducted to a depth of $N_{d}=10^{3},$ with a sample being collected from the population every generation. Systematic sampling of evolving populations is becoming increasingly feasible (Good *et al.* 2017); here, a very thorough sampling of the system was used to grant a clearer comparison of the different inference methods. To test the effect of a sparser sampling regime, a range of simulations were repeated with data collected to a depth of $N_{d}=10^{2}$ every 10 generations, that is, at $t_{k}=0,10,20,\ldots .$ Our model simulates the effect of strong selection, with Ns≫1, but is not restricted to the strong mutation paradigm of μN≫1 (Rouzine *et al.* 2001; Park *et al.* 2010). In each case, the initial state of the system was defined by $n_{0}\left(0\right)=N$ and $n_{i}\left(0\right)=0$ for all i>0.

### Inference methods

Inferences of selection were conducted using an evolutionary model to generate inferred haplotype frequencies $q_{i}\left(t\right)$ across time. Given observations of the system, a multinomial log likelihood was calculated for the system

$$L\left({\left\{q_{i}\left(t_{k}\right)\right\}}_{k=1}^{K}\right)=\sum_{k=1}^{K}\log\frac{N_{d}!}{\prod_{i=0}^{L}o_{i}\left(t_{k}\right)!}\prod_{i=0}^{L}{\left(q_{i}\left(t_{k}\right)\right)}^{o_{i}\left(t_{k}\right)}$$ (3)

where $o_{i}\left(t_{k}\right)$ is the number of observations of the haplotype *i* at the time $t_{k},$ and the sum is calculated over data from all observed time points. Three models were used to generate inferred frequencies. In each model, the inferred frequencies are generated by the fitness parameters $s_{i}$ (in the case of the linear system, by the single parameter *s*) and by the initial haplotype frequencies $\left\{q_{i}\left(0\right)\right\},$ with an additional parameter *β* being required for the delay-deterministic model. Parameters were optimized to identify the maximum log likelihood.

#### Deterministic inference model:

In the first model, haplotype frequencies were modeled under the assumption of an infinite population size. As such, in each generation a fraction of each haplotype was subject to mutation, specified by the function *M*:

$$M\left(q_{i}\left(t\right)\right)=\left(1-\mu \right)q_{i}\left(t\right)+\mu \sum_{j}q_{j}\left(t\right)$$ (4)

where the sum was conducted over all haplotypes *j* that differ from *i* by a single allele, giving mutation between adjacent haplotypes as illustrated in Figure 1. Selection was included in a similarly deterministic manner, with each haplotype increasing or decreasing in frequency according to its fitness, specified by the function *S*:

$$S\left(q_{i}\left(t\right)\right)=\frac{w_{i}q_{i}\left(t\right)}{\sum_{j=0}^{L}w_{j}q_{j}\left(t\right)}$$ (5)

where the sum was calculated over all haplotypes. The next generation is given by

q(t+1)=S(M(q(t))) (6)

#### Stochastic inference model:

In the second model, allele frequencies were propagated in exactly the same way as in the model used for simulation. Stochastic simulations of viral populations have been used to explore the potential range of outcomes occurring in viral systems (Russell *et al.* 2012). To sample the space of potential outcomes, 1000 replicates of the model were run for each set of initial parameters *s* and $\left\{q_{i}\left(0\right)\right\},$ generating 1000 sets of inferred frequencies $q_{i}\left(t\right).$ The mean value of the likelihoods for these replicates was then computed, the likelihood for each replicate being calculated using Equation 3. A simple likelihood maximization approach was used in the optimization; to account for the stochasticity of the likelihood function, the optimization routine was prevented from resampling previously tested model parameters.

#### Delay-deterministic model:

Finally, a delay-deterministic model was implemented, identical to the deterministic model described above, but with the addition of a delay representing the time for establishment of individuals with a novel haplotype. Specifically, the mutation function of Equation 4 was modified, with mutation out of a haplotype occurring only if the frequency of that haplotype was greater than a specific threshold. Accordingly, mutation was modeled via the new function $M^{\prime }:$

$$M^{\prime }\left(q_{i}\left(t\right)\right)=\left(1-I_{i}\left(t\right)\mu \right)q_{i}\left(t\right)+\mu \sum_{j}I_{j}\left(t\right)q_{j}\left(t\right)$$ (7)

where the index function

$$I_{i}\left(t\right)=\{\begin{array}{ll} \;1: & q_{i}(t)\geq \beta \\ 0: & otherwise \end{array}$$ (8)

The parameter *β* was optimized to identify the maximum likelihood model.

We note that, of the three inference models, the stochastic model requires an estimation of the total population size, *N*; for the sake of computational time, we used the correct value of this parameter in our inferences. Neither the deterministic or delay-deterministic models require an estimate of population size.

#### Application to experimental data:

To explore the use of our approach with experimental data, the deterministic and delay-deterministic models were applied to influenza sequence data collected from an evolutionary experiment in which the transmission of a reassortant H5N1 influenza virus was observed between pairs of ferrets (Wilker *et al.* 2013). In this experiment, the evolution of the virus was observed using genome sequence data generated from samples collected from the inoculum and from directly infected index ferrets 1, 3, and 5 days after infection, and from the contact ferrets, infected via transmission, 7 and 9 days after contact with the index ferrets. A previous analysis of these data using a deterministic model inferred that, during the course of the experiment, new viral haplotypes, generated via mutation, grew in frequency under very strong positive selection (Illingworth 2015), matching the essential characteristics of the model system considered above. Here, deterministic and delay-deterministic models were applied to within-host data from a single animal in the study, denoted F3501 in the original work, for which the initial population diversity was relatively low. Genome sequence data from the hemagglutinin segment of the virus were processed using the method described in a previous publication (Illingworth 2015), identifying loci at which significant change in allele frequency was observed, then processing short-read data spanning these loci into a set of multi-locus variant calls and inferring haplotype frequencies that best fit the observed data using a maximum likelihood model. Mutation was initially modeled as occurring deterministically between haplotypes, identifying an optimal model of haplotype fitness using a model selection procedure, whereby the most parsimonious explanation of the data was calculated using the Bayesian Information Criterion (BIC) (Kass and Raftery 1995). Fitness parameters within this model were then reinferred using the delay-deterministic framework, comparing fitnesses inferred using each approach. In common with the default model in the original study, the rate of mutation was modeled as $\mu =10^{-5},$ using an assumed generation time of 12 hr (Baccam *et al.* 2006). We note that this system departs from the linear arrangement of haplotypes used in the simulated systems; our simulations are intended to show where differences between the different models arise.

### Data availability

Code used for this work is available at https://github.com/cjri/delaydet. The authors state that all data necessary for confirming the conclusions presented in the article are represented fully within the article.

## Results

### Linear fitness landscape

**Figure 2:**
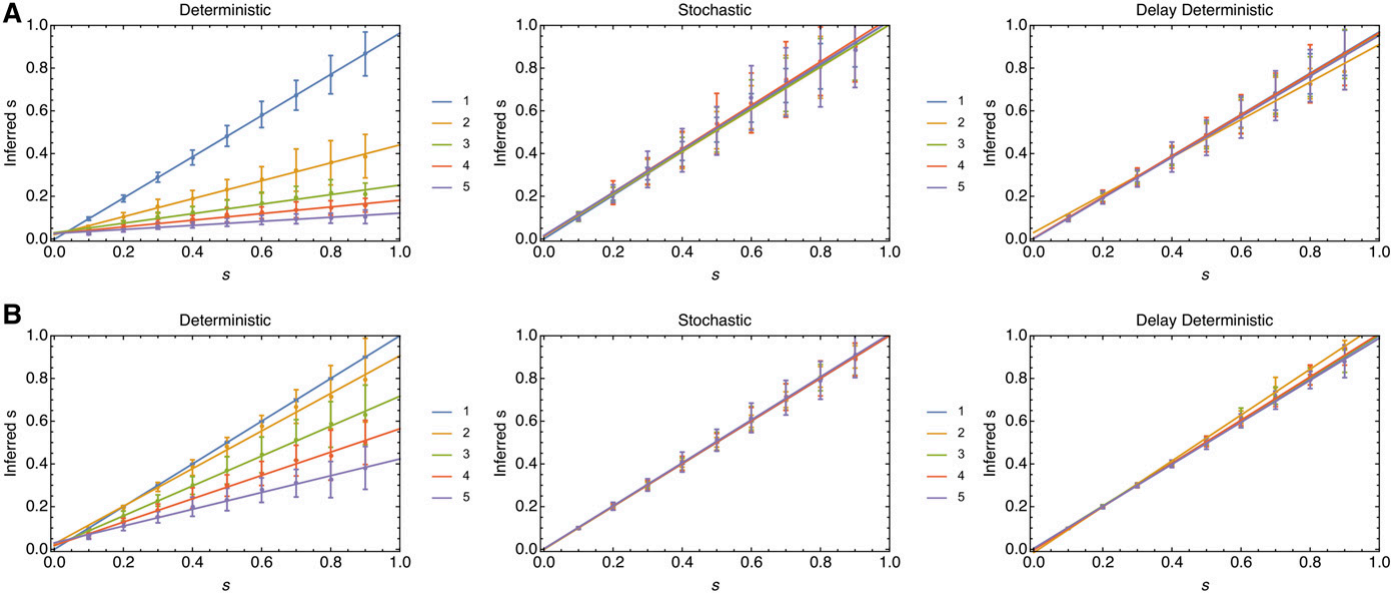
The deterministic model substantially underestimates the correct magnitude of selection for models with multiple haplotypes. Parameters are shown for values of *L* between 1 and 5 with (A) μN=1. (B) μN= 10,000. Points show mean inferred selection coefficients; error bars were determined from a set of 100 replicate calculations. Lines show the outputs of a linear regression model fitted to the mean values.

In the simulations modeling a linear fitness landscape, the stochastic and delay-deterministic models produced the most accurate inferences of selection. Selection coefficients inferred from the three different models are shown in Figure 2. For each of the values of μN, the deterministic model underestimates the magnitude of selection, *s*, for values of *L* greater than 1 (that is, where there were three or more haplotypes in total), with an increasing degree of underestimation as *L* increases. Where L=5, the gradient of a linear regression model fitted to the mean inferred frequencies was equal to 0.095; roughly one-tenth of what would be given by a correct inference model. The results obtained also depend upon the value of μN; where this statistic is larger, the extent to which the deterministic model underestimated the true magnitude of selection was reduced; here, for the case, L=5 the gradient of the fitted linear model was 0.39. Results from the stochastic inference model show a good reproduction of the correct fitness values; linear gradients varied between 0.98 and 1.02 for the case μN=1, indicating an accurate reproduction of selection coefficients as would be expected given the identical models of propagation. The delay-deterministic method was close in performance to the stochastic model, with gradients between 0.88 and 0.97 indicating a small underestimate of the strength of selection. This underestimate was not reproduced in the case μN= 10,000, for which gradients fitted to the delay-deterministic outputs were either side of 1. Results from our downsampled data set showed a slight increase in the variance of fitness estimates, but the fundamental pattern of results was preserved (Supplemental Material, Figure S1 in File S1). An analysis of the likelihoods produced under this sampling paradigm showed that the delay-deterministic model was favored under BIC for systems with L≥2 (Figure S2 in File S1).

**Figure 3:**
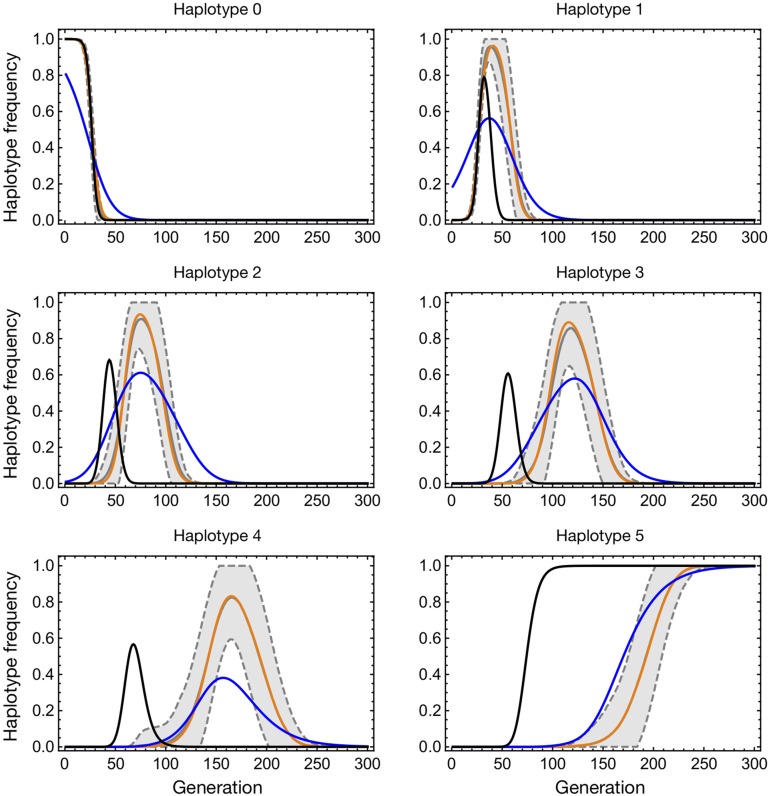
Time-dependent dispersion of trajectories for the case μN=1, L=5, and s=0.5. Frequencies of each haplotype are shown reading left to right from the top. In each case, the solid gray line (sometimes obscured) shows the mean haplotype frequency of the simulated data across time, calculated across 100 simulations. The region within 1 SD of this frequency is indicated by gray dashed lines and is shaded. The black line shows the propagation of the deterministic model in the case where s=0.5 and the population starts at the same haplotype distribution as the simulation, while the blue line shows the results of a maximum likelihood fit between the deterministic model and the mean data. The mean maximum likelihood fit of the delay-deterministic model to the data are shown as an orange line.

The results that we obtained can be intuitively understood via a plot of the evolutionary dynamics of the linear system (Figure 3). Given a deterministic model with the correct selection coefficient, the population propagates through the haplotypes substantially faster than does the stochastic model. In the Wright–Fisher model, given that Nμ=1, a mean of one individual mutates from haplotype 0 to haplotype 1 in the first generation. Following the second generation, the probability of an individual being found in haplotype 2 is therefore approximately *μ*. In so far as double mutations are ignored within our model framework, at least one individual is required to occupy a haplotype before the next haplotype can be founded via mutation; this leads to a delay of multiple generations before a single individual reaches the final haplotype, following which selection ensures the eventual fixation of this haplotype. By contrast, in the deterministic model, mutation propagates the population rapidly through the system; after *L* generations, the final haplotype is deterministically occupied by a frequency of the population of order $\mu^{L}.$ The increased fitness of this final haplotype therefore takes effect on the system more rapidly, leading to the observed faster propagation. When the deterministic model is optimized, a lower fitness parameter *s* is inferred to compensate for this effect, which increases dependent upon the number of haplotypes in the system. By contrast, the delay-deterministic model corrects for the error of the deterministic model. By imposing a delay on the rate at which new haplotypes are founded by mutation, the rate of propagation through the haplotypes is controlled, giving an improved fit to the data and therefore a more accurate inference of selection.

At higher values of μN, differences between the stochastic and deterministic systems are reduced (Figure S3 in File S1). As *N* tends to infinity, the number of individuals mutating between haplotypes per generation approaches the deterministic limit and the time at which a haplotype becomes established decreases, with the consequence that less of a reduction in *s* is required to fit the model to the data. The deterministic model therefore provides a good description of the behavior of the discrete system as $\mu^{L}N$ in the discrete model approaches a value much larger than 1. For a within-host model of influenza, where *μ* may be of the order $10^{-4}$ (Pauly *et al.* 2017), and *N* potentially as large as $10^{14}$ (Russell *et al.* 2012), this implies that a value of *L* ≥ 4 could lead to failure of the deterministic model.

**Figure 4:**
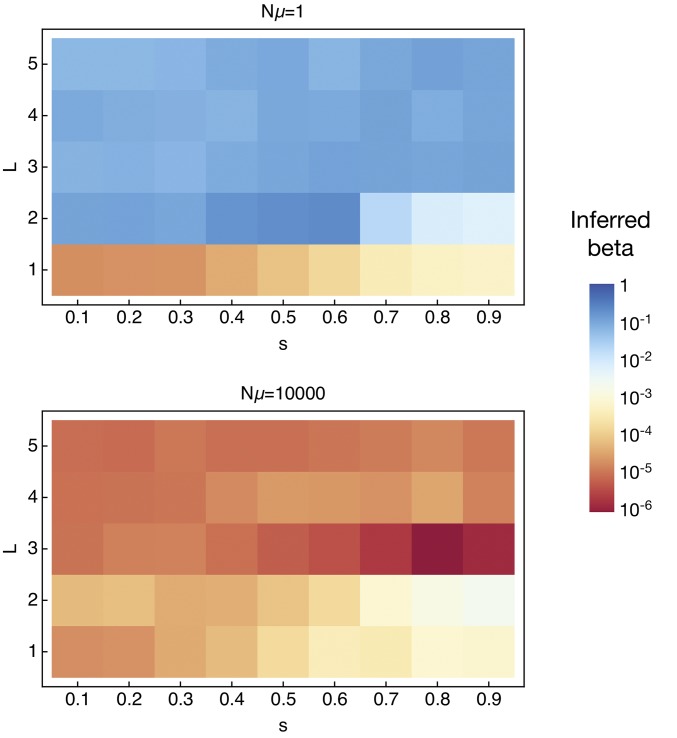
Median values of the inferred parameter *β* from the delay-deterministic model for simulations conducted with Nμ=1. Each value is calculated from 100 simulations. Considerable variance was observed in the optimized parameter for each set of simulations.

Within the delay-deterministic model fits, a broad range of values of the inferred parameter *β* were obtained, spanning several orders of magnitude (Figure 4). For cases in which L>1 and Nμ=1, where the behavior of the deterministic model is furthest from that of the simulated population, quite large values of *β* were inferred, with a range in the median inferred values from 0.5 to 21%. Much smaller values of *β* were inferred where L=1 or $N\mu =10^{4}.$ For each of these cases, the deterministic model provides a better approximation of the dynamics of the system; the deterministic model may be considered as a special case of the delay-deterministic model for which β=0. As such, a smaller value of *β* was generally inferred. Substantial variation in the inferred value of *β* was observed between replicate simulations generated with the same parameters (Figure S4 in File S1). This suggests that the generation of an analytic approximation for this value is likely to prove a challenge.

### Convex and concave fitness landscapes

**Figure 5:**
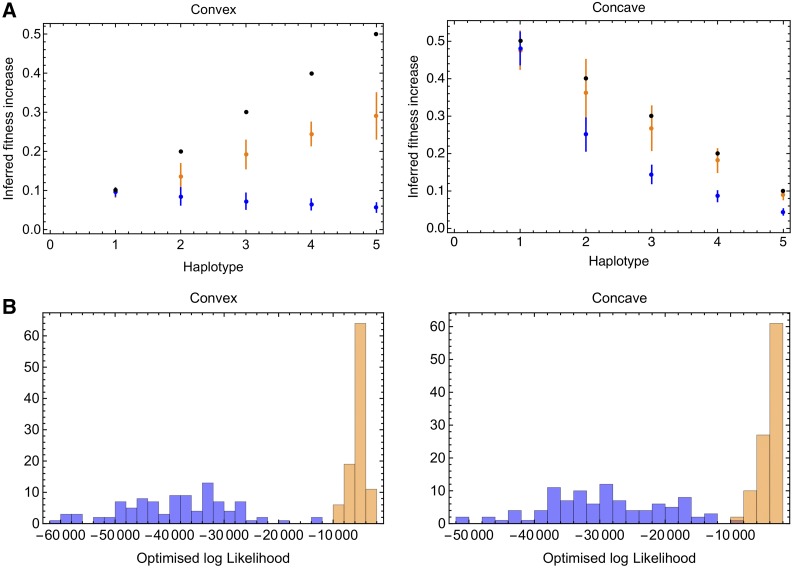
Inferences from convex and concave fitness landscapes. (A) Fitness differences between haplotypes are shown as black dots. Mean and SD of inferred fitness differences were calculated for 100 replicates in each case, and are shown for the deterministic (blue) and delay-deterministic (orange) methods. (B) Optimized log likelihoods, describing the fit between the model and the data obtained for the deterministic (blue) and delay-deterministic (orange) methods.

Application of the deterministic and delay-deterministic models to data from the convex and concave fitness landscapes showed an improved inference of selection coefficients in the case of the delay-deterministic methods (Figure 5A). However, in contrast to the calculations for the linear fitness landscape, the delay-deterministic method showed substantial deviation from the correct selection coefficients. We propose that this arises from the mechanics of the emergence of haplotypes. The time to the emergence of a new haplotype is dependent upon the gain in fitness obtained by this transition, and in this case differs between pairs of haplotypes. The delay-determininstic method only has a single parameter with which to model this, so produced an approximation to the correct result. While an imperfect solution, the inclusion of a delay parameter granted a substantially better reproduction of the dynamics of the system. In so far as the deterministic model is a special case of the delay-deterministic model, the maximum likelihood fit of the delay-deterministic model can never be lower than that of the deterministic model. Nevertheless, the likelihood fits obtained for these two systems showed considerable differences between likelihoods (Figure 5B). We propose that where variation exists in the fitness differences between adjacent haplotypes, a model in which independent values of *β* were fitted to each haplotype could give a better fit to the data, albeit with a concurrent cost in the time taken to optimize individual parameters; more advanced delay-deterministic approaches were not investigated in this study.

### Within-host influenza evolution

**Figure 6:**
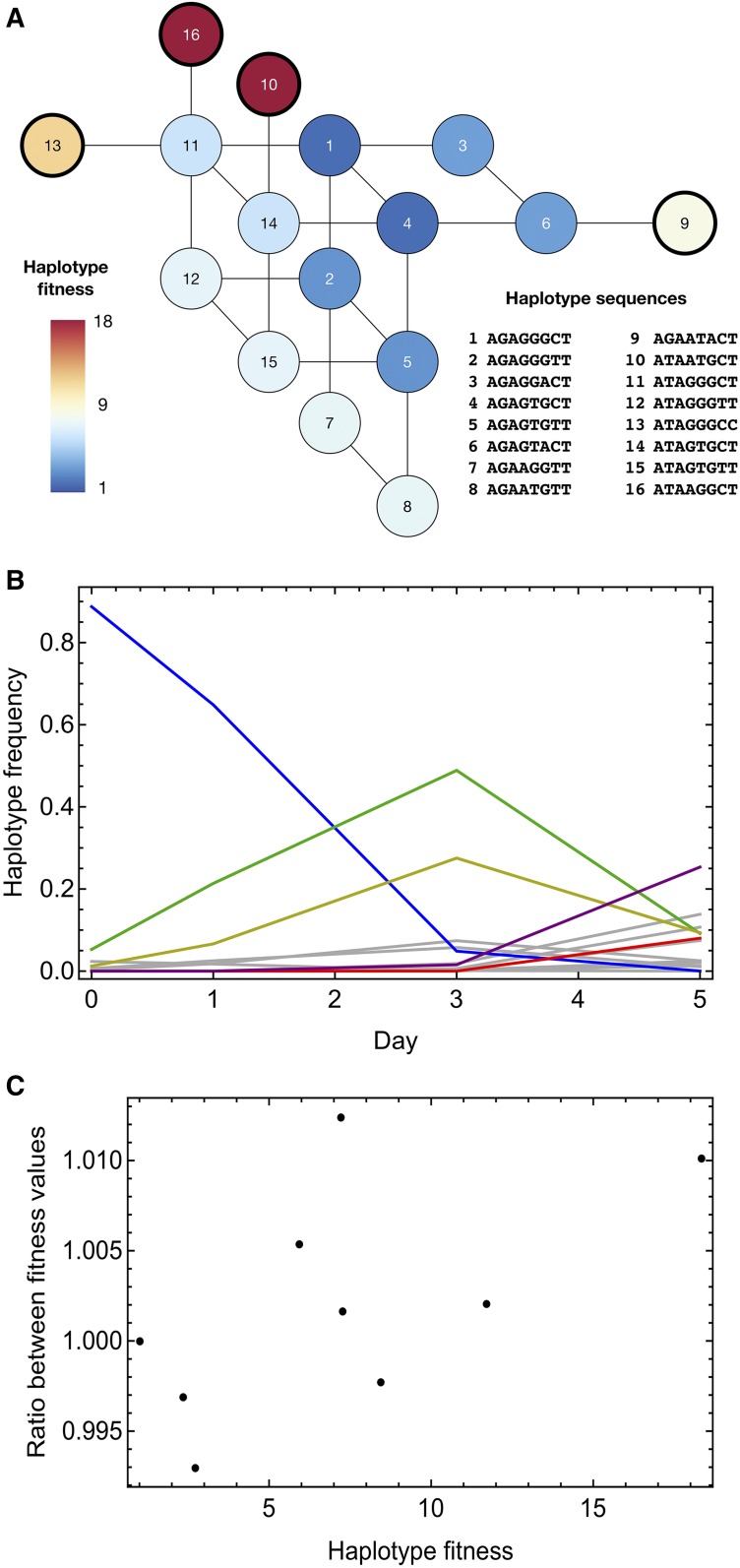
(A) Fitness landscape inferred from experimental data using the deterministic method. Inferred fitnesses are given for haplotypes for which the inferred frequency reached ≥ 1% within the course of within-host evolution. Given haplotypes describe the sequence of the hemagglutinin segment of the virus at genome positions 339, 496, 728, 738, 788, 1018, 1020, and 1144. The haplotypes 9, 10, 13, and 15, marked in darker outline, were inferred to have zero initial frequencies. (B) Inferred changes in haplotype frequency over time. Haplotype frequencies are shown in gray, with the exceptions of haplotypes 1 (blue), 2 (green), 6 (yellow), 12 (purple), and 16 (red). (C) Differences in inferred haplotype fitness values between the deterministic and delay-deterministic methods shown proportional to the value inferred under the deterministic model.

Application of the deterministic and delay-deterministic methods to data from an evolutionary experiment (Wilker *et al.* 2013) showed only small differences between inferred parameters. Details of each inference are given in Table S1 in File S1. Within this system evolution proceeds exceptionally fast, with mutation into new and highly advantageous haplotypes being inferred to drive the adaptation of the system over the course of an infection (Figure 6, A and B). Application of the delay-deterministic value gave a marginally improved fit to the data, with a maximum likelihood value 0.63 units better than the deterministic model without accounting for the additional parameter; under the BIC this does not represent a significant improvement in the model. The value of *β* was inferred to be $1.8\times 10^{-10},$ smaller than that of any inferred initial haplotype frequency that was greater than zero. However, the inferred haplotype fitness values were very similar between the models, with deviations in fitness of not much more than 1% (Figure 6C). This final result can be understood in terms of the arrangement of haplotypes within the system; although some haplotypes were inferred to have initially zero frequency, being created by mutation from other haplotypes, there was insufficient time for haplotypes that were two or more mutations away to increase to an appreciable frequency. This result is informative for calculations performed on biological data sets; even where selection for novel variants is extreme in nature, a delay-deterministic model is unlikely to be required to generate correct inferences of selection on timescales of 4–5 days (∼10–20 generations). This result implies that previous inferences of selection for within-host influenza adaptation using deterministic methods are unlikely to be negatively affected by the use of a deterministic model of mutation (Sobel Leonard *et al.* 2017). Rather, the value of the method will arise over longer timescales, where the population grows under selection into haplotypes that are separated by multiple variants from those that comprise the initial population.

## Discussion

Deterministic models of adaptation have been proposed as a rapid and effective method for inferring selection from time-resolved sequence data (Jewett *et al.* 2016). Here, we have highlighted a limitation of such frameworks whereby a deterministic model may severely underestimate the magnitude of selection in a system. This underestimation results from delays in the propagation of a finite population toward mutationally distant haplotypes; at least one individual is required to occupy a haplotype before mutation out of that haplotype may occur. As shown here, as this delay operates even at high values of Nμ; we suggest that a population size satisfying $N\mu^{L}\gg 1$ would be required to remove this effect. As a solution to this problem, we propose an alternative inference procedure, which we term a delay-deterministic model. Under this model, the progress of a nominally infinite population through the system is delayed via the use of an additional model parameter, bringing the outcome closer to the behavior of the stochastic system. As such, relative to a regular deterministic model, an improved inference of selection is obtained; in the case of a linear fitness landscape, correct inferences were obtained.

In demonstrating the application of our model, we have chosen the simplest possible situation in which the effects we are studying apply; that of a linear set of haplotypes separated by single mutations. Such a system, with a linear fitness landsacpe, has previously been considered in an application to cancer, calculating the time at which a novel haplotype might arise (Beerenwinkel *et al.* 2007); while the system we consider is similar, our research question differs from this earlier study. We note that our model is not the only approach that would give a correct inference of selection under the circumstances of a population entering mutationally distant haplotypes. For example, inferring an “establishment time” for each haplotype, at which a haplotype enters a population at a frequency above the selection–drift threshold (Illingworth and Mustonen 2012), would likely generate correct results, albeit at the cost of learning a single parameter per haplotype in the system. The use of a model of time-dependent selection, in which selection only begins to affect a haplotype at a specific point in time (Kessinger *et al.* 2013), would also give an approximately correct inference of selection, delaying the impact of selection to a point at which the inferred trajectory would fit the data. However, this again would incur a computational cost and would require the imposition on the system of the potentially incorrect assumption of a change in the magnitude of selection with time. Approximations to the stochastic system based upon a reproduction of the stochastic distribution of allele frequencies (Martin and Lambert 2015) may have some potential for evolutionary inference, albeit this has not to our knowledge been attempted. The delay-deterministic model we present here gives a computationally rapid approach to infer the magnitude of selection, improving upon the accuracy of the regular deterministic approach.

In comparison with the stochastic model of inference, the delay-deterministic approach has the computational advantage of utilizing a framework of deterministic propagation. Whereas the stochastic model required a large number of replicate propagations of the model for each set of parameters tested, the delay-deterministic approach requires only the optimization of a single additional parameter. The relative cost of this is likely to vary considerably depending upon the complexity of the system in question; in the application to influenza data considered here, where the model contained tens of parameters to be optimized, a single additional parameter is not likely to add substantially to the computational cost, provided that the optimization procedure is implemented in an efficient manner. We note that faster implementations of the stochastic framework are likely to be achievable; the recent demonstration of such methods for the two-allele case suggest the extension to more generalized population models to be a valuable avenue for exploration (Khatri 2016; Krukov *et al.* 2017).

Applying our model to data from an evolutionary experiment, we identified very similar results between the deterministic and delay-deterministic methods; despite very high magnitudes of selection being inferred to act upon haplotypes in this case, little difference in the model inferences was found. Therefore, we propose that our method will be of relevance in cases where selection is strong and acts over longer time periods than those of the experiment considered, for which adaptation was observed over only a small number of generations. The identification of cases for which the deterministic model will produce correct or incorrect results is likely to be possible via application of the model itself; wherever a substantial proportion of a population is inferred to evolve into a haplotype that is more than two mutations distant from a haplotype occupied by the initial population, a delay-deterministic or similar approach should be considered.

## Supplementary Material

Supplemental material is available online at www.genetics.org/lookup/suppl/doi:10.1534/genetics.118.300790/-/DC1.
