# Supplementary material for: Inferring Fitness Effects from Time-Resolved Sequence Data with a Delay-Deterministic Model
Source: Genetics. 2018 Mar 2;209(1):255–64. doi: 10.1534/genetics.118.300790 (PMC5937181; doi:10.1534/genetics.118.300790)
Supplement: Supplementary file 1 [file 255FileS1.pdf]

A delay-deterministic model for inferring fitness effects from time-resolved genome sequence data

Nuno R. Nené, Alastair S. Dunham, Christopher J. R. Illingworth
Department of Genetics, University of Cambridge, Cambridge, UK

Supporting Tables

Supporting Table 1 Parameters inferred with the deterministic and delay-deterministic models for the influenza sequence data.

Parameter	Deterministic model	Delay-deterministic model
Likelihood	-1326.51	-1325.88
σ_{339}	-1.098	-1.041
σ_{496}	1.773	1.778
σ_{738}	1.125	1.130
σ_{1018}	1.011	1.004
σ_{1020}	0.858	0.855
σ_{1144}	0.687	0.684
$\chi_{496,1018}$	-1.651	-1.657
$\chi_{1018,1020}$	-0.779	-0.771
$\chi_{496,1020}$	-0.671	-0.660
$\chi_{496,738,1020}$	-1.927	-2.075
β		1.79×10^{-10}

Supporting Figures

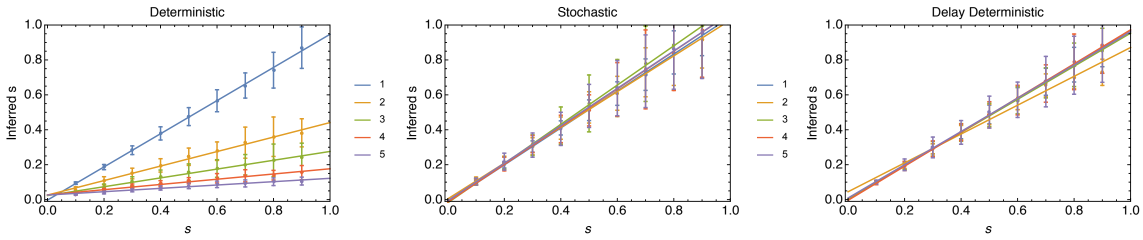

Figure S1. Results from a downsampled set of simulations, containing 1% of the amount of data of the default simulations. Parameters are shown for values of L between 1 and 5 with $\mu N = 1$. Points show mean inferred selection coefficients; error bars were determined from a set of 100 replicate calculations. Lines show the outputs of a linear regression model fitted to the mean values

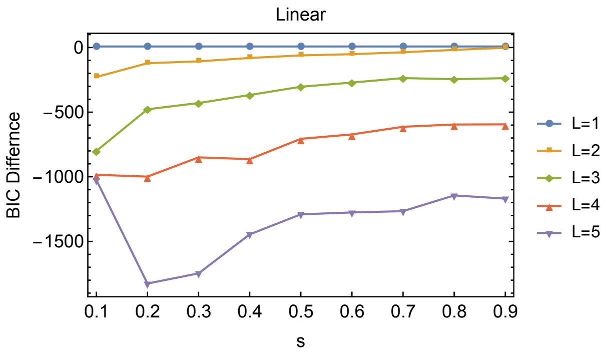

Figure S2. Median BIC differences between the deterministic and delay-deterministic models applied to the downsampled simulation data. Parameters are shown for values of L between 1 and 5 with $\mu N = 1$. A negative BIC difference indicates that the delay-deterministic approach was the better model; this is the case for all values with $L \geq 2$, but no values with $L = 1$. We note that the optimisation code is not guaranteed to find the global maximum likelihood for the system for every dataset considered.

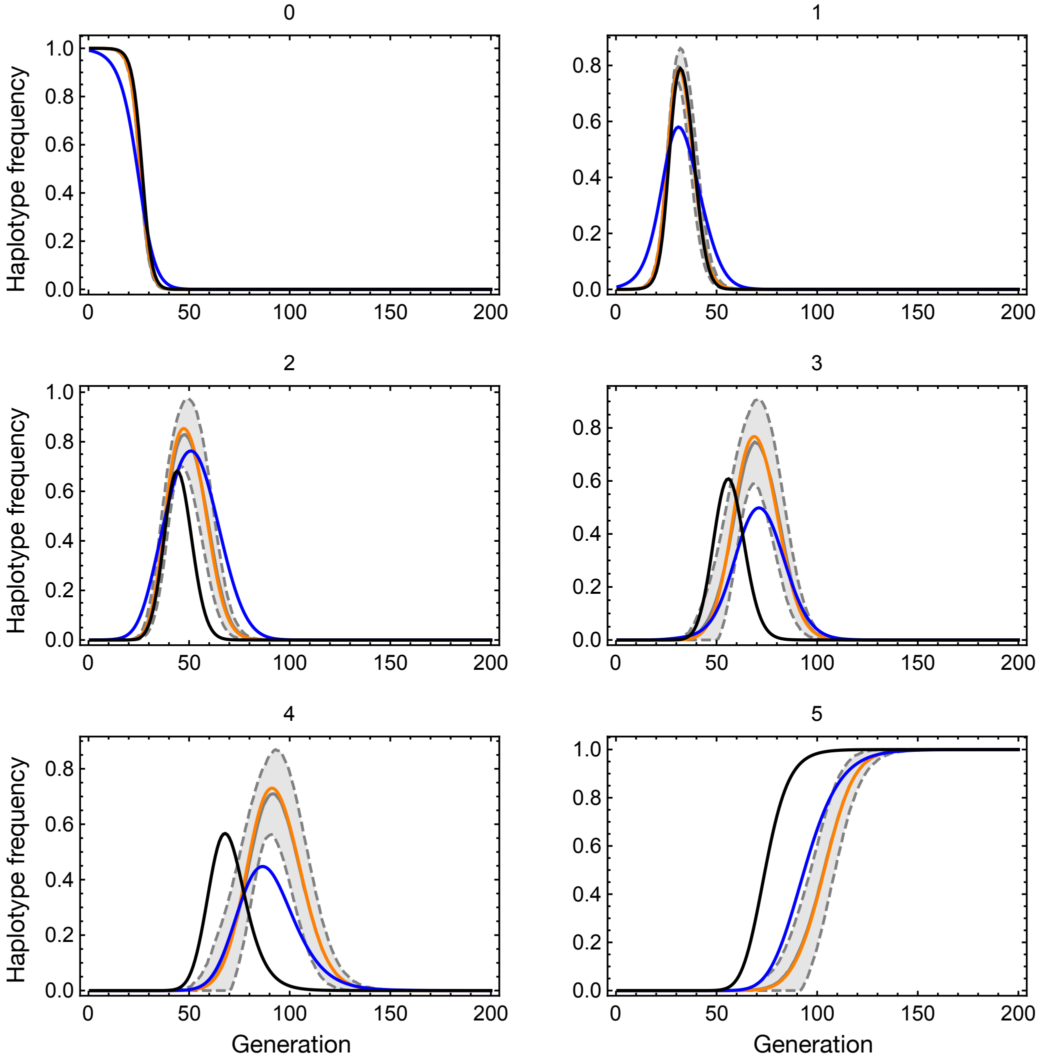

Figure S3. Time-dependent dispersion of trajectories for the case $\mu N = 10000$, $L = 5$ and $s = 0.5$. Frequencies of each haplotype are shown reading left to right from the top. In each case the solid gray line shows the mean haplotype frequency of the simulated data across time, with the variation in this frequency across 100 simulations shown by the gray region around this. The black line shows the propagation of the deterministic model in the case where $s = 0.5$, while the blue line shows the results of a maximum likelihood fit between the deterministic model and the mean data. The maximum likelihood fit of the delay-deterministic model to the data is shown by the orange line.

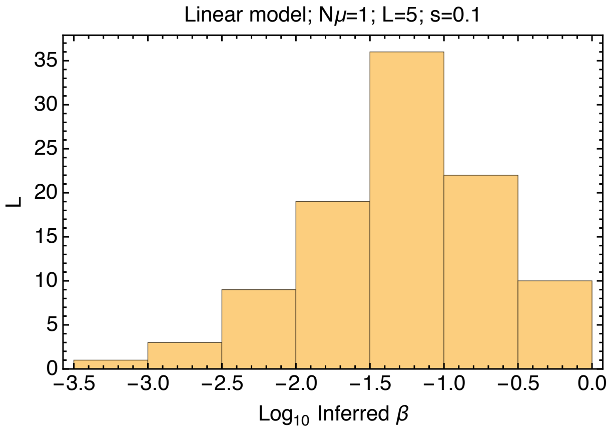

Figure S4. Inferred values of the parameter β from the delay-deterministic model for replicate simulations conducted for the linear fitness landscape with the parameters $N\mu = 1$, $L = 5$, and $s = 0.1$. Inferred values of β range from 5.2×10^{-4} to 0.71.
